# Paravertebral block for surgical anesthesia of percutaneous nephrolithotomy: Care-compliant 3 case reports

**DOI:** 10.1097/MD.0000000000004156

**Published:** 2016-07-18

**Authors:** Yong Liu, Xiao Yu, Xingxing Sun, Qing Ling, Shaogang Wang, Jihong Liu, Ailin Luo, Yuke Tian, Wei Mei

**Affiliations:** a Department of Anesthesiology; b Department of Urology, Tongji Hospital, Tongji Medical College, Huazhong University of Science and Technology, Wuhan, China.

**Keywords:** case report, paravertebral block, percutaneous nephrolithotomy, surgical anesthesia

## Abstract

**Background:**

Paravertebral block is often used to provide postoperative analgesia after renal surgery. In this case-series report, we present our experience with 3 patients in whom percutaneous nephrolithotomy was performed successfully under ultrasound-guided 3-segment lumbar-thoracic paravertebral block.

**Case summary:**

Three patients were scheduled for percutaneous nephrolithotomy. All 3 patients were high-risk cases for both general and neuraxial anesthesia. After due deliberation and with the consent of patient and his family, ultrasound-guided paravertebral block was performed. Seven to 10 mL of 0.5% ropivacaine was injected at T10/T11, T11/T12, and T12/L1 paravertebral place, respectively. Sensory loss to pinprick from T8 to L2 was achieved in all 3 patients 20 min after administration of block. Surgical procedures for all 3 patients were successful, and none of the patients complained of pain during the operation.

**Conclusions:**

Ultrasound-guided multilevel paravertebral block may be an attractive option for anesthetic management of percutaneous nephrolithotomy in clinical practice.

## 1 Introduction

Percutaneous nephrolithotomy (PCNL) can be safely performed under both general and neuraxial anesthesia. However, in some special situations, both general and neuraxial anesthesia are associated with increased risk of complications, or are contraindicated. Anesthesia management in these patients is a real challenge. Paravertebral block (PVB) is used to provide effective postoperative analgesia after many urological procedures.^[1]^ With meticulous planning and use of refined block technique under ultrasound guidance, PVB can achieve adequate somatic and visceral sensory blockade to provide anesthetic cover for PCNL. In this case-series report, we present our experience with use of ultrasound-guided 3-segment PVB to provide surgical anesthetic cover for PCNL in 3 patients. The reporting of these cases was approved by the Ethics Committee at the Tongji Hospital, Tongji Medical College, Huazhong University of Science and Technology. Written informed consent was obtained from all 3 patients.

## 2 Case descriptions

### 2.1 Case 1

A 60-year-old man with a 3 × 3 cm calculus in the left renal pelvis was scheduled for percutaneous holmium laser lithotripsy. He weighed 65 kg and was 172 cm tall. He had a history of mitral valve-replacement surgery and was on warfarin therapy. He also had a history of asthma for more than 20 years with severe pulmonary decompensation. International normalized ratio (INR) at admission was 2.26. He was shifted from warfarin to low molecular weight heparin therapy. INR decreased to 1.23 on the day prior to the operation. Because of pulmonary decompensation and the need for intensive anticoagulation therapy, he was a high-risk case for both general anesthesia and neuroaxial block. After careful discussion with multidisciplinary team and due deliberation and with the consent of patient and his family, PCNL was performed under ultrasound-guided PVB.

### 2.2 Case 2

A 67-year-old man with multiple calculi in the right renal pelvis and calyces was scheduled for PCNL. His weight and height were 60 kg and 163 cm. He had a history of stroke sustained 6 months prior to admission, which resulted in hemiplegia. He also had a history of chronic obstructive pulmonary disease. Preoperative pulmonary function tests revealed severe obstructive ventilatory impairment. Preoperative electrocardiogram indicated atrial fibrillation. 24-h Holter monitoring revealed long RR interval (>3 seconds, 3 times). Temporary pacemaker was implanted before the operation. This patient was at increased risk for general anesthesia because of poor pulmonary function. He had a relative contraindication to neuroaxial block because of hemiplegia. Based on our successful experience with PVB in the previous patient, we decided to adopt the same technique for this patient. With the consent of patient and his family, ultrasound-guided PVB was performed. PCNL was successfully performed with minimal interference with cardiopulmonary function.

### 2.3 Case 3

A 27-year-old man with staghorn calculi was scheduled to undergo a second PCNL. His weight and height were 70 kg and 172 cm. He had a history of lumbar vertebral fracture and had a relative contraindication to neuroaxial block. He had undergone PCNL under general anesthesia one week earlier. Postoperatively, he experienced severe nausea and vomiting. He refused to accept general anesthesia this time. With the agreement of patient, we chose ultrasound-guided PVB for him.

## 3 Ultrasound-guided PVB

On arrival at the operation room, the patient was placed in a supine position. Standard intraoperative monitoring with electrocardiography, pulse oximetry, and noninvasive blood pressure measurement was performed. Hydromorphone 1 to 2�mg was administered intravenously 10 minutes prior to the nerve block for patient's comfort during puncture. The patients were turned to lateral position with the side to be operated upward. Oxygen was administered by facemask. The level of nerve block planned for the 3 patients was T10/T11, T11/T12, and T12/L1, respectively.

**Figure 1:**
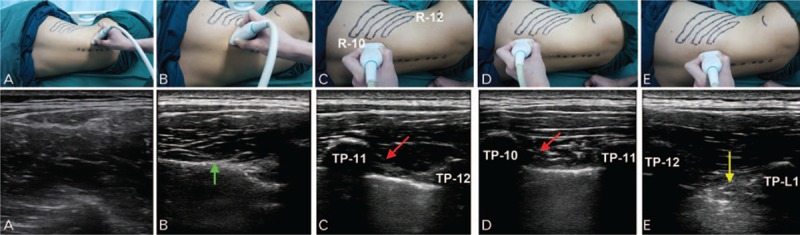
Illustration of the step-wise technique for identification of the level of transverse process. A, The probe is placed at the lumbar back parallel to the rib. a, The lumbar muscles are seen on the ultrasonogram. B, The probe is parallel-shifted cephalad. b, The 12^th^ rib (green arrow) comes into view. C, The probe is rotated to the sagittal plane. c, The transverse process of T11 and T12 is in the same visual field, and the superior costotransverse ligament (red arrow) is identified. D, The probe is moved cephalad. d, Transverse process of T10 and T11 is in the same visual field. E, The probe is moved caudad. e, transverse process of T12 and L1 is in the same visual field, and the intertransverse ligament (yellow arrow) is identified. R = rib, TP = transverse process.

Ultrasound-guided PVB was performed using a linear array US 5- to 10-MHz probe (Mindray, Shenzhen, China). The probe was positioned parallel to the rib through the lumbar back. The orientation marker was directed caudolaterally to generalize transverse slice (Fig. 1A). The lumbar muscles were easily identifiable (Fig. 1A). The probe was moved cephalad parallel to the rib (Fig. 1B) until a rib (12th rib) was visualized (Fig. 1B). At that point, the probe was rotated anticlockwise to generalize sagittal slice of the rib. The probe was moved medially and the transverse process of T 12 and T 11 identified (Fig. 1C). With the probe being moved cephalad or caudad, the transverse process of T 10 (Fig. 1D) or L 1 (Fig. 1E) was also identified. The T10/T11, T11/T12, and T12/L1 paravertebral spaces were chosen for injection in the 3 cases, respectively.

For PVB, an “off side” approach was used as described by Abdallah.^[2]^ After infiltration of 2% lidocaine, a 100-mm, 21-gauge Tuohy needle (SonoPlex Nanoline; Pajunk Inc, Geisingen, Germany) was introduced in a caudad-to-cephalad direction by in plane technique under direct visualization of the needle tip. Seven to 10 mL of 0.5% ropivacaine was injected at each segment.

Under cystoscopic guidance, a ureteral stent was placed with use of topical anesthesia (5% lidocaine gel). The patient was then place in a prone position for operation.

## 4 Results

Sensory loss to pinprick from T8 to L2 was achieved in all 3 patients 20 minutes after administration of block (Table 1). None of the patients complained of pain during the establishment of percutaneous tract, distension of the renal capsule and pelvicaliceal system, lithotomy and during placement of nephrostomy tubes. No additional opioids or nonsteroidal anti-inflammatory drugs were needed during the operation. The duration of surgery was 40, 100, and 136 minutes, respectively. Surgical procedures for all 3 patients were successful, and none of the patients required blood transfusion. Details of the procedures are summarized in Table 2.

**Table 1:**
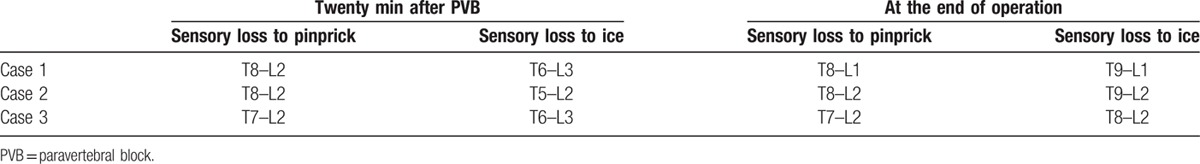
Assessment of sensory block achieved in the 3 patients.

**Table 2:**
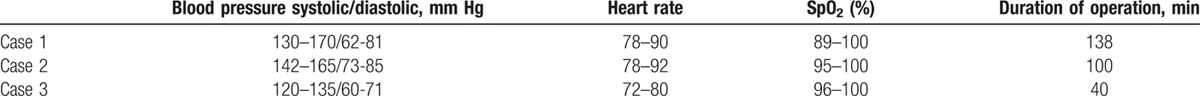
Intraoperative parameters.

## 5 Discussion

All 3 patients in this case series were high-risk cases for general as well as neuraxial anesthesia. Under PVB, all 3 patients underwent PCNL successfully; the intraoperative period was uneventful. PVB has been successfully used for postoperative analgesia after urological surgery.^[1]^ However, its use to provide anesthetic cover for PCNL has not been documented. Achievement of adequate analgesia for PCNL requires blockage of both somatic nerves that innervate skin and muscle tissue, and the visceral nerves that innervate kidney and the ureters. The tract for lithotripsy is typically established in the 10th to 11th intercostal space, or in the subcostal area.^[3]^ Sensory nerves in this area are readily blocked by thoracic PVB. Renal pain is thought to be transmitted via nerves originating from T10 to L1. The ureter is innervated by nerves originating from T10 to L2.^[4]^ Based on this anatomical knowledge, complete blockade of unilateral spinal nerves from T10 to L2 can provide sufficient analgesia during PCNL.

The thoracic paravertebral space is continuous over all thoracic levels through the adipose tissue at the anteromedial corner.^[5]^ However, the communication between thoracic and lumbar paravertebral spaces has been the subject of debate. Some have disputed the existence of a communication between thoracic and lumbar paravertebral space owing to the presence of psoas major that is believed to completely seal off the paravertebral space at the lumbar level.^[6]^ In a study, dye injected into the thoracic paravertebral place did not spread caudally to the L1 lumbar segment.^[6,7]^ However, several other studies appear to suggest otherwise.^[8–10]^ For example, local anesthetics injected at the thoracic paravertebal space have been shown to lead to sensory blockade of lumbar spinal nerves.^[8,9]^ In order to ensure complete blockade from T10 to L2, we injected at both thoracic and lumbar paravertebral spaces separately.

Confining the injection to a single thoracic level may compromise the extent of somatic blockade.^[9,11]^ In all 3 cases, we separately injected local anesthetic at the respective 2 target segments (T10/T11, T11/T12, and T12/L1).

Thoracic PVB is convenient to administer ultrasound guidance. However, little guidance is available on the technique for ultrasound guided lumbar PVB in the published literature. Usually, it is guided by loss of resistance or stimulator guidance.^[12,13]^ In our clinical setting, we usually employ ultrasound for lumbar PVB. The key point in the procedure is to identify the intertransverse ligament (Fig. 1E), as the anesthetic agent is required to be injected under it.

For PVB, the level of vertebrae is usually identified by palpation,^[7,11]^ although it may not always be accurate. To locate the exact level of the transverse process, we developed a novel method for identification of the desired vertebral level. Using this method, we first identify the 12th rib and the 12th thoracic vertebrae, for which 3 key points need to be considered: from caudad to cephalad, the 12th rib is the first rib to come into view; the 12th rib disappears on being traced laterally; and moving caudally from the T12 vertebrae, L1 can be distinctly identified from thoracic vertebrae because of lack of any connected rib. We find this methodology convenient to use.

Every 3 to 4 mL of local anesthetic induces loss of somatic sensation in 1 dermatome on thoracic PVB.^[14,15]^ Therefore, 7 to 10 mL of 0.5% ropivacaine was injected at each segment. Ultimately, all 3 patients achieved sensory block at least from T8 to L2, which proved to be adequate for the operation.

Some limitations of our report need to be acknowledged. We arbitrarily chose a 3-segament PVB using 7 to 10 mL of local anesthetic agent. Multiple punctures may lead to patient discomfort as well as increase the risk of pleural puncture. It needs to be clarified whether a 2-segment or even 1-segment PVB can be as effective as 3-segament PVB. If so, which level/levels is/are most suitable is another question. The optimal requirement of local anesthetic agent is also not clear. Further, the tract for lithotripsy may be different based on the location of the calculi. Therefore, choice of the segment/levels for PVB should be chosen individually. In addition, we did not perform continuous paravertebral nerve block for postoperative analgesia. In our hospital, PCNL is always completed with 2 h. Single PVB with 0.5% ropivacaine is known to provide analgesic cover for more than 24 h,^[15]^ which is adequate for intra- and postoperative analgesia. Lastly, PCNL is a minimally invasive surgery typically associated with only moderate postoperative pain. Therefore, patients may be easily managed with oral nonopioid or opioid analgesics.

In conclusion, this is the first report of multilevel PVB performed to provide anesthetic cover for PCNL. This technique is an attractive alternative for patients who are at an increased risk of postoperative complications following general or neuraxial anesthesia.
